# Characterization of a GDP-Fucose Transporter and a Fucosyltransferase Involved in the Fucosylation of Glycoproteins in the Diatom *Phaeodactylum tricornutum*

**DOI:** 10.3389/fpls.2019.00610

**Published:** 2019-05-21

**Authors:** Peiqing Zhang, Carole Burel, Carole Plasson, Marie-Christine Kiefer-Meyer, Clément Ovide, Bruno Gügi, Corrine Wan, Gavin Teo, Amelia Mak, Zhiwei Song, Azeddine Driouich, Patrice Lerouge, Muriel Bardor

**Affiliations:** ^1^Bioprocessing Technology Institute, Agency for Science, Technology and Research (A^∗^STAR), Singapore, Singapore; ^2^Laboratoire Glyco-MEV EA4358, UNIROUEN, Normandy University, Rouen, France; ^3^Fédération de Recherche Normandie-Végétal – FED 4277, Rouen, France; ^4^Institut Universitaire de France (I.U.F.), Paris, France

**Keywords:** diatom, fucosylation, nucleotide-sugar transporter, *Phaeodactylum tricornutum*, glycosylation, Golgi apparatus, fucosyltransferase, biopharmaceuticals

## Abstract

Although *Phaeodactylum tricornutum* is gaining importance in plant molecular farming for the production of high-value molecules such as monoclonal antibodies, little is currently known about key cell metabolism occurring in this diatom such as protein glycosylation. For example, incorporation of fucose residues in the glycans *N*-linked to protein in *P. tricornutum* is questionable. Indeed, such epitope has previously been found on *N*-glycans of endogenous glycoproteins in *P. tricornutum*. Meanwhile, the potential immunogenicity of the α(1,3)-fucose epitope present on plant-derived biopharmaceuticals is still a matter of debate. In this paper, we have studied molecular actors potentially involved in the fucosylation of the glycoproteins in *P. tricornutum.* Based on sequence similarities, we have identified a putative *P. tricornutum* GDP-L-fucose transporter and three fucosyltransferase (FuT) candidates. The putative *P. tricornutum* GDP-L-fucose transporter coding sequence was expressed in the Chinese Hamster Ovary (CHO)-gmt5 mutant lacking its endogenous GDP-L-fucose transporter activity. We show that the *P. tricornutum* transporter is able to rescue the fucosylation of proteins in this CHO-gmt5 mutant cell line, thus demonstrating the functional activity of the diatom transporter and its appropriate Golgi localization. In addition, we overexpressed one of the three FuT candidates, namely the FuT54599, in *P. tricornutum* and investigated its localization within Golgi stacks of the diatom. Our findings show that overexpression of the FuT54599 leads to a significant increase of the α(1,3)-fucosylation of the diatom endogenous glycoproteins.

## Introduction

Diatoms are marine organisms that represent one of the most important source of biomass in the ocean (Nelson et al., 1995; Raven and Waite, 2004; Bowler et al., 2008). There has been a surge in developing the use of diatoms as a source of bioactive compounds in the food and cosmetic industries (Spolaore et al., 2006; Mata et al., 2010; Cadoret et al., 2012). In addition, the potential of diatoms such as *Phaeodactylum tricornutum* (*P. tricornutum*) as solar-powered cell factories for the production of biopharmaceuticals has been demonstrated (Mathieu-Rivet et al., 2014; Hempel and Maier, 2016). For instance, *P. tricornutum* has been used to produce monoclonal antibodies (mAbs) (Hempel et al., 2011, 2017; Hempel and Maier, 2012). These alga-made mAbs are either directed against the highly pathogenic Marburg virus, which belongs to the same family as Ebola virus (Hempel et al., 2017) or the Hepatitis B virus surface antigen (Hempel et al., 2011; Hempel and Maier, 2012). Both recombinant mAbs produced in *P. tricornutum* were demonstrated to be able to recognize and bind their respective antigen. In addition, the mAb directed against the Hepatitis B was demonstrated to be of good quality, homogenous and glycosylated with oligomannosides (Vanier et al., 2015). This mAb is also able to bind to human Fcγ receptors (FcγRI and FcγRIIIa in particular) which suggests that it could be efficiently used in human immunotherapy to induce phagocytosis and antibody dependent cell-mediated cytotoxicity response (Vanier et al., 2018). Such therapeutic application represents currently a multimillion dollar market sales (Walsh, 2014). However, when compared to a human IgG1 used as a control, affinity of the diatom-made mAb is 4.5-fold lower than the one of the human IgG1 for FcγRI and three-times higher for FcγRIIIa. Such differences in kinetics and affinity are due to *N*-glycosylation variance (Vanier et al., 2018). Therefore, it would be necessary in the future to engineer the *N*-glycosylation of diatom-produced mAb to favor the presence of complex-type and fine-tuned *N*-glycans as it is well established that glycosylation of mAbs and biopharmaceuticals in general influences their biological functionality and efficacy (Lingg et al., 2012; Buettner et al., 2018; Mimura et al., 2018).

In this context, production of therapeutic proteins dedicated to human therapy in *P. tricornutum* requires a comprehensive understanding of the glycosylation biosynthesis that operates in the diatom. For instance, fucosylation of glycans *N*-linked to biopharmaceuticals produced in *P. tricornutum* is questionable. Indeed, glycosylation analysis of endogenous proteins demonstrated the presence of paucimannosidic glycans bearing an α(1,3)-fucose (Baïet et al., 2011). Moreover, putative immunogenicity of proteins produced in plants has been reported to be due to α(1,3)-fucose epitopes introduced by the plant expression system (Wilson et al., 1998; Bardor et al., 2003; Scha ahs et al., 2007). Such glyco-epitopes are absent in mammalian cells and thus could be immunogenic when proteins carrying such decorations are injected into mammals (van Beers and Bardor, 2012). This question is still a matter of debate. Indeed, previous study demonstrated the presence of antibodies raised against plant α(1,3)-fucose in 25% of non-allergic blood donors over 53 sera (Bardor et al., 2003). Another study reporting a phase I clinical trial for a plant-derived vaccine demonstrated that only 7 out of 48 volunteers (14.6%) had detectable amount of IgG directed against plant *N*-glycans including the α(1,3)-fucose epitope (Landry et al., 2010). More recently, Ward et al. (2014) reported that 19.2% of the subjects were positive for IgG antibodies directed against plant glyco-epitopes prior to vaccination and that 34% of the vaccinated volunteers developed IgG, and eventually IgE responses to plant glyco-epitopes after vaccination, even if no allergic/hypersensitivity response was observed. Recently, the taliglucerase alpha, the first plant cell-expressed biotherapeutic was approved on the market and is currently used for Enzyme Replacement Therapy to treat Gaucher Disease (Fox, 2012). This approved biopharmaceutical is a glycoprotein bearing as a major exposed glycan (representing more than 90% of the glycoforms) a paucimannosidic *N*-glycans substituted by an α(1,3)-fucose and a β(1,2)-xylose (Shaaltiel et al., 2007; Tekoah et al., 2013). In a Phase I clinical trial in healthy human volunteers, the injection of the taliglucerase alpha did not induce obvious adverse side effects that could be attributed to the plant *N*-glycan glyco-epitopes (Aviezer et al., 2009; Rup et al., 2017).

Fucosylation of glycoproteins requires the cytosolic biosynthesis of GDP-L-fucose and its import into the Golgi cisternae prior to its transfer onto the glycoproteins through the action of Golgi-localized fucosyltransferases (FuT). As mentioned earlier, biochemical investigation of the protein *N*-glycosylation in *P. tricornutum* has demonstrated that endogenous proteins carry mainly oligomannosides and little amount of paucimannosidic-type *N*-glycans carrying a fucose residue α(1,3)-linked to the proximal *N*-acetylglucosamine (GlcNAc) residue (Baïet et al., 2011). Moreover, three putative FuT have been predicted in the genome of *P. tricornutum* (Baïet et al., 2011; Mathieu-Rivet et al., 2014). In the present paper, we report on the characterization of molecular actors involved in the fucosylation of glycans *N*-linked to *P. tricornutum* proteins. This includes, in addition to the FuT candidates, the identification of a sequence encoding homolog of a putative GDP-L-fucose transporter (PtGFT). The later has been cloned and expressed in the Chinese Hamster Ovary (CHO)-gmt5 mutant cell line, a mammalian cell line deficient in GDP-L-fucose transporter activity (Zhang et al., 2012; Haryadi et al., 2013). We show that PtGFT is able to rescue the fucosylation of proteins in the CHO-gmt5 mutant cell line, thus demonstrating the functional activity of the diatom transporter. To the best of our knowledge, PtGFT represents the first microalgae nucleotide-sugar transporter to be functionally characterized so far. Moreover, we demonstrate that FuT54599 (encoded by the Phatr3_J54599 gene) candidate is localized in the Golgi apparatus in *P. tricornutum*. Finally, we found that overexpression of the FuT54599 leads to an increase of the α(1,3)-fucosylation of the endogenous glycoproteins from *P. tricornutum*.

## Materials and Methods

### Culture of *P. tricornutum*

The *P. tricornutum* strain Pt1.8.6 (CCAP1055/1) was grown in reconstituted artificial seawater (AQUARIUM SYSTEMS Instant Ocean) enriched with Conway medium containing 80 mg.L^−1^ of sodium metasilicate (Na2SiO3), at 19 ± 1°C as described previously in Ovide et al., 2018. The culture were grown under a 16 h/8 h light/night cycle (280–350 μmol photons m^−2^⋅s^−1^) and agitation at 150 rpm.

*Phaeodactylum tricornutum* cells expressing the V5-tagged FuT54599 or the V5-tagged GnT I were grown in F/2 medium containing 1,5 mM NH4Cl as nitrogen source and no sodium metasilicate, under a 16 h/8 h light/night cycle (280–350 μmol photons m^−2^⋅s^−1^) and agitation at 150 rpm for the first 4 days at 19 ± 1°C and then under continuous illumination (280–350 μmol photons m^−2^⋅s^−1^) for the next 5 days at 23°C ± 1°C. Liquid cultures were grown with a 150 rpm agitation in a volume of 150 mL. For the expression of the V5-tagged glycosyltransferases, cells were induced at day 6 by transferring cells in a fresh F/2 medium containing 0.9 mM NaNO_3_ as nitrogen source according to (Hempel and Maier, 2012).

### Monosaccharide Composition Analysis of *P. tricornutum* Fractions

*Phaeodactylum tricornutum* cell pellets were resuspended in 70% ethanol with lysing beads (D-matrix lysing tubes, MP Biomedicals^®^) and ground for 6 cycles during 30 s at 6.5 m.s^−1^ in a FastPrep-24^TM^ homogenizer (MP Biomedicals^®^). Crushed cells were incubated at 70°C for an hour. Extractions were then performed to remove lipids from the cell wall fraction. Briefly, the residues were extracted once with methanol: chloroform (1: 1 v/v), then with acetone at room temperature under agitation. Residues were dried under pure air flush. The monosaccharide composition of this alcohol insoluble residue (AIR) was analyzed by gas chromatography coupled to a Flame Ionization Detector spiking inositol as an internal standard as described previously (Louvet et al., 2011). One mg of each fraction was hydrolyzed in 2 M trifluoroacetic acid during 2 h at 110°C. Trifluoroacetic acid was washed twice with a 50% *iso*-propanol: water solution. The released monosaccharides were converted to their *O*-methylglycosides by incubation in 1 M methanolic HCl at 80°C overnight (Moore et al., 2006). After evaporation of methanol and HCl, the methyl-glycosides were resuspended in 200 μL of a methanol: pyridine mixture (4: 1 v/v) then submitted to a re-*N*-acetylation reaction by adding 50 μL of pure Acetic Anhydride and incubated for 1 h at 110°C. Re-*N*-acetylated samples, after evaporation of reagents were then converted into their trimethylsilyl derivatives by heating the samples for 20 min at 110°C in hexamethyldisilizane: trimethylchlorosilane: pyridine (3: 1: 9 v/v/v). After evaporation of the reagent, the samples were washed twice and finally suspended in 1 mL of cyclohexane before being injected in a CP-Sil 5 CB column (Agilent Technologies, United States). Data were integrated with the GC Star Workstation software (Varian/Agilent Technologies, United States). A temperature program (3 min at 40°C; up to 160°C at 15° min^−1^; up to 220°C at 1.5° min^−1^; up to 280°C at 20° min^−1^; 3 min at 280°C) was optimized for the separation of the most common cell wall monosaccharides. The GC-FID analyses were ran in triplicate on extracts isolated from 4 independent cell cultures.

### Bioinformatic Analyses

#### Database Search, Protein Sequences Alignments and Phylogenetic Analysis

Search for putative *P. tricornutum* GFT coding sequences was carried out by BlastP (2.2.28) searches (Altschul et al., 1997) in the sequence data of *P. tricornutum* in the Ensembl Protists database (release 40 – July 2018 EMBL-EBI). The topology of the potential PtGFT was predicted using the TMHMM (Sonnhammer et al., 1998) and Phoebius (Käll et al., 2004) tools.

Comparison of the protein sequences of various eukaryotic GFT was performed using the MUSCLE program (Edgar, 2004). This includes sequences of GFT which has been already functionally characterized (Luhn et al., 2001, 2004; Geisler et al., 2012; Peterson et al., 2013; Rautengarten et al., 2016) such as the one from *Homo sapiens* (NP_060859.4; *SLC*35C1 gene), *Mus musculus* (NP_997597.1; *SLC*35C1 gene), *Cricetulus griseus* (NP_001233737.1; *SLC*35C1 gene), *Caenorhabditis elegans* (NP_001263841.1; nstp-10 gene), *Drosophila melanogaster* (NP_649782.1; Dm_Gfr gene and *Arabidopsis thaliana* (NP_197498.1; At5g19980 (GFT1/GONST4) genes.

Structure analysis of the putative *P. tricornutum* FuT and various characterized FuT from plant and invertebrates was done by using the NCBI Conserved Domain Database (Marchler-Bauer et al., 2017) and Pfam database (El-Gebali et al., 2019) tools. Comparison of the amino acid sequences corresponding to the Glyco_Transf_10 domains described in the NCBI Conserved Domain Database was carried out by the T-coffee web server^1^ (Di Tommaso et al., 2011).

A phylogenetic tree was built with the GOLGI-LOCALIZED NUCLEOTIDE SUGAR TRANSPORTER GONST1, GONST2, GONST3, and GFT1/GONST4 amino acids sequences of *A. thaliana* (At2g13650, At1g07290, At1g76340, and At5g19980 genes), respectively (Baldwin et al., 2001; Handford et al., 2004; Mortimer et al., 2013), the *P. tricornutum* coding sequences corresponding to the Phatr3_J43174, Phatr3_J45630, and Phatr3_J9609 genes, similar to the GONST sequences and the functionally characterized animal GDP-L-fucose transporters from *H. sapiens* (NP_060859.4, SLC35C1_GDP-fucose transporter 1 isoform a), *C. elegans* (NP_001263841.1, GDP-fucose transporter), *D. melanogaster* (NP_649782.1, Gfr) and also the *H. sapiens* SLC35C2 (NP_001268386, solute carrier family 35 member C2 isoform d). The phylogenetic tree was drawn using the *Phylogeny.fr* platform (Dereeper et al., 2008, 2010) using the “One click” mode. The analysis used follow three steps: (i) complete sequences were aligned with MUSCLE 3.8.31 (Edgar, 2004); (ii) ambiguous regions (i.e., containing gaps and/or poorly aligned) were removed with Gblocks (v0.91b) (Castresana, 2000); and (iii) the phylogenetic tree was built using the maximum likelihood method implemented in the PhyML program (v3.0 aLRT) (Guindon et al., 2010). Graphical representation and edition of the phylogenetic tree were performed with TreeDyn (v198.3) (Chevenet et al., 2006). Finally, the phylogenetic tree viewer PhyD3 was used to finalize the figure^2^ (Kreft et al., 2017).

### Cloning of the V5-Tagged FuT54599 and the GnT I Coding Sequences for Overexpression

The GnT I-V5 insert was obtained from a plasmid construct containing the GnT I coding sequence (GenBank: HM775384.1) fused to the V5-tag of the pcDNA3.1/V5-His-TOPO vector (described in Baïet et al., 2011) by PCR amplification with the Phusion^TM^ high-fidelity DNA polymerase (Finnzymes) and the following forward primer 5′-CAATTGATGCGGTTGTGGAAACG-3′ and reverse primer 5′-GGATCCTCTTTTCGGTGACGGAA-3′. After purification, the PCR product was cloned in the pJET1.2/blunt (Thermo Fisher), verified by Sanger sequencing and then inserted as a MunI- BamHI restriction fragment in the pPha-NR expression vector (GenBank accession number: JN180663) digested with the EcoRI and HindIII restriction enzymes (Thermo Scientific). Transformation of *P. tricornutum* Pt1.8.6 cells was done by biolistic as described by Hempel and Maier (2012). The positive transformants were selected by PCR analysis as described below. The cloning of the V5-tagged-FuT54599 sequence in the pPha-NR vector was carried out by the same way excepted that the V5-tagged-FuT54599 was obtained by gene synthesis (according to the genomic sequence of the Phatr3_J54599 gene, GeneCust). The primers used to retrieve the V5-tagged-FuT54599 insert by PCR amplification were 5′-GAGCTCATGTCACTTCGCAAG-3′(forward) and 5′-AAGCTTACGTAGAATCGAGACCGAGGAGA-3′ (reverse). Finally, the FuT54599-V5 coding sequence was inserted in the pPha-NR as a SacI-HindIII restriction fragment before transforming *P. tricornutum* cells.

### PCR and RT-PCR Analysis

For DNA or RNA isolations, sub-culturing of *P. tricornutum* cells was conducted in two 500 mL Erlenmeyer flasks containing 200 mL of sterilized fresh medium. At steady state (1 × 10^8^ cells.mL^−1^), the cells were pelleted by centrifugation at 4,500 g during 10 min, at 4°C and then resuspended in 1 mL of NucleoZOL (Macherey-Nagel, GmbH & Co. KG, Düren, Germany) for RNA isolation or in the lysis buffer PL1 (Macherey-Nagel) for DNA extraction. Then, samples were transferred in lysing matrix E, 2 mL tubes (MP Biomedicals^®^), immediately frozen in liquid nitrogen and stored at −80°C until purification. Cell lysis was carried out by using the FastPrep-24^TM^ homogenizer (MP Biomedicals^®^) for 4 cycles of 30 s, at 6.5 m.s^−1^. Then, after 5 min of incubation at room temperature and a centrifugation step of 5 min at 12,000 *g*, the supernatant was recovered and transferred to a new 2 mL tube. Genomic DNA was purified with the Nucleospin Plant II kit (Macherey-Nagel) according to the manufacturer’s instructions. Total RNA was isolated using a combination of the NucleoZOL reagent method (Macherey-Nagel) for the extraction and the NucleoSpin RNA Plus kit (Macherey-Nagel) for purification following the supplier’s instructions. After DNase treatment with the TURBO DNA-*free*^TM^ Kit (Invitrogen^TM^), the first-strand cDNA was synthetized from 2 μg of RNA using the High-Capacity cDNA Reverse Transcription Kit with RNase Inhibitor (Applied Biosystems^TM^).

The PCR reactions were prepared according to the GoTaq^®^ G2 DNA Polymerase protocol (Promega) in a total volume of 20 μL. A 2 μL aliquot of a 1:10 dilution of gDNA or cDNA was added to the mixture and, in parallel, a reaction with 2 μL of water was prepared as a negative control. PCR was performed in a Veriti Thermal Cycler (Applied Biosystems^TM^) using a 3 steps program as follow: 5 min of initial denaturation at 95°C, followed by 35 cycles of 30 s for denaturation at 95°C, 30 s for annealing at 60°C, 30 s for extension at 72°C and a final elongation for 5 min at 72°C. A 14 μl aliquot of the PCR reaction was analyzed on a 1.8% agarose gel stained with SafeView^TM^ (ABM) to reveal the amplified products.

A primer pair specific to the putative PtGFT and allowing to distinguish the cDNA and DNA sequences was designed with the Primer-Blast program (Ye et al., 2012) using the nucleotide sequence NCBI accession number XM_002177440.1 as the template. The forward primer 5′-TTGTCGGGCATCTTCTGGTC-3′ and the reverse primer 5′-GACGAATTCCCAGGCACGTA-3′ were used in this work.

To screen the *P. tricornutum* cells transformed with the V5-tagged GnT I coding sequence by PCR amplification of DNA, the same primer pair as the one used for retrieving the sequence from the pcDNA3.1/V5-His-TOPO vector (described in the section “Cloning of the V5-Tagged FuT54599 and the GnT I Coding Sequences for Overexpression” of the Materials and Methods) was chosen.

For the screening of the *P. tricornutum* transformants expressing the Phatr3_J54599 gene fused to a 3′ V5-Tag a forward primer specific to the FuT54599 coding sequence (5′-GCCAGGCCAATTATAGTCGC-3′) was used in combination with a reverse primer specific to the V5-tag (5′-GACCGAGGAGAGGGTTAGGG-3′).

### Complementation of the CHO-gmt5 Line

The coding sequence of the candidate PtGFT was used to prepare a DNA construct in the pcDNA^TM^3.1^(+)^ Mammalian Expression Vector (Invitrogen, Life Technologies). The PtGFT coding sequence from the NCBI accession no. XM_002177440.1 (nucleotides 39–1121) in fusion with the nucleotide sequence encoding a HA-tag at its 5′ end and a “GCCACC” Kozak sequence was obtained by gene synthesis and then cloned as a *Hind*III-*Xho*I fragment in the pcDNA^TM^3.1^(+).^plasmid. The synthetic DNA sequence is registered under the NCBI accession number KT737477. Transient expression of PtGFT gene in CHO-gmt5 cell line, immunodetection of proteins and affinostaining with *Aleuria Aurantia Lectin* (AAL), were carried out as previously reported in Zhang et al. (2012).

### *N*-Glycan Profiling of CHO-gmt5 Proteins

1 × 10^7^ CHO cells at the mid-exponential phase (day 4) were harvested, washed 3 times with PBS 1X and resuspended in 1 mL of extraction buffer (25 mM Tris, 150 mM NaCl, 5 mM EDTA, 1% CHAPS, pH 7.4) prior to sonication for 15 min. The samples were then centrifuged at 500 *g* for 10 min. The supernatant was saved whereas the pellet was extracted a second time using 500 μL of the same extraction buffer. The second supernatant was pooled with the previous one before dialyzing against 4 × 1 L of 50 mM ammonium bicarbonate, pH 8.5 at 4°C for 24 h using 7000 MWCO dialysis cassette. After 24 h of dialysis, the sample was transferred to a 7 mL Teflon-lined capped amber glass vial. 2 mL of 50 mM Tris-HCl, pH 8.5 containing 2 mg.mL^−1^ of dithiothreitol was added to the sample. After homogenization, the sample was incubated in the dark at 37°C for 1 h under rotation at 20 rpm. Iodoacetic acid (10 mg.mL^−1^) was added to the sample, vortexed and incubated at 37°C for another 2 h in the dark. At the end, this carboxymethylation process was terminated by dialyzing the sample against 4 × 1 L of 50 mM ammonium bicarbonate, pH 8.5 at 4°C for 24 h. The sample was then transferred to a 7 mL Teflon-lined capped glass vial and finally evaporated to dryness. The reduced carboxymethylated proteins were digested with 40 μg of trypsin (Promega) and further deglycosylated by peptide-*N*-glycosidase F (PNGase F) (Prozyme). The digestions, purification and permethylation of the resulting *N*-glycans were performed as previously described in Yusufi et al. (2017). MALDI-TOF mass spectrometry data was acquired on a 5800 MALDI-TOF/TOF mass spectrometer (AB Sciex, Foster City, CA, United States) in positive reflectron mode. Permethylated samples were reconstituted in 30 μL of 80% (v/v) methanol in water. 0.5 μL of the sample was then spotted on a target plate along with 0.5 μL of matrix [10 mg.mL^−1^ 2,5-dihydroxybenzoic acid (Water Corporation, Milford, MA, United States) dissolved in 80% (v/v) methanol in water]. The 4700 calibration standard kit, calmix (AB Sciex) was used as the external calibrant for the MS mode. The mass spectrum of the sample was acquired from a mass range of *m/z* 500–5,000 with total accumulated shots of 10,000. The laser intensity used was 80%.

### Transmission Electron Microscopy

High pressure freezing, freeze substitution and transmission electron microscopy of *P. tricornutum* expressing V5-tagged FuT54599 or GnT I were carried out as described in Ovide et al. (2018). Immunocytochemistry of V5-tagged transferases was carried out using antibodies raised against the V5 epitope (mouse anti-V5 tag antibodies, Invitrogen, dilution 1/20) and second antibody (EM-goat anti mouse IgG + IgM 10 nm gold particles, BBI solution, dilution 1/20). A classical staining using uranyl acetate/lead citrate and eventually KMnO_4_ was done before observation as previously described (Venable and Coggeshall, 1965).

### Extraction of Proteins for Western Blot Analysis

The expression of the V5-tagged FuT54599 or GnT I was induced at day 6 by transferring *P. tricornutum* cells in a fresh 100% seawater medium containing 0.9 mM NaNO_3_. After 24, 48, and 72 h, cell cultures were harvested by centrifugation (2,000 *g* during 10 min). Cell pellets were frozen. The cell pellets were then re-suspended in a 0.1 M Tris buffer pH7 containing a Protease Inhibitor Cocktail (SIGMAFAST^TM^ Protease Inhibitor Cocktail Tablets, EDTA-Free) and the cells were broken down using the FastPrep-24^TM^ homogenizer (MP Biomedicals^®^) as described for PCR and RT-PCR analysis. The extracted proteins were centrifuged first at 10,000 *g* during 30 min giving the total protein extract. Sample was then centrifuged at 100,000 *g* during 1 h 30 at 4°C.

Pellet containing the membrane proteins was separated from the intracellular proteins that were present in the supernatant. Both fractions were analyzed by NuPAGE Bis-Tris Gel electrophoresis and Western Blot analysis. About 50 μg of intracellular proteins were denatured with the Laemmli sample buffer during 10 min at 100°C and loaded on a NuPAGE Bis-Tris gel (4–12%). Likewise, membrane fractions were loaded on the gel after denaturation. The migration of the proteins through the gel was carried out in a MOPS buffer at 180 V during 1 h. For the detection of V5-tagged glycosyltransferases such as the FuT54599 or the GnT I, 1 μg of *Escherichia coli* Positive Control (*E. coli*) Whole Cell Lysate (Abcam) was used as a positive control (presence of the V5-tag). Five μL of the PageRuler Plus Prestained Protein Ladder (Thermo Fisher) was loaded as molecular weight markers. After separation on the NuPAGE Bis-Tris gel, proteins were blotted on a nitrocellulose membrane using a semi-dry transfer Thermo Scientific^TM^ Pierce^TM^ Power Blotter. A Ponceau S staining was performed to visualize the efficiency of the protein transfer on the nitrocellulose membrane.

For the detection of the V5-tagged FuT54599, the nitrocellulose membrane was saturated overnight in TBS-T buffer and then incubated with a primary rabbit anti-V5 antibody (Invitrogen) at a dilution of 1/3,000 in TBS-T for 2 h at room temperature. The secondary antibody used was a goat anti-rabbit antibody conjugated with HRP (Sigma). It was used at a dilution of 1/30,000 in TBS-T for 1 h at room temperature. Revelation was performed using the ECL west Pico plus kit (Thermo Fisher) according to the manufacturer’s instructions. Exposure time was 1 min.

Above 50 μg of total protein extract were loaded on a NuPAGE Bis-Tris gel (4–12%) after denaturation and the migration of the proteins through the gel was carried out in a MOPS buffer as described above. One μg of PLA_2_ from honey bee venom (14.5 kDa, SIGMA-ALDRICH) was used as a positive control and 1 μg of Ribonuclease B from bovine pancreas (15 kDa, SIGMA-ALDRICH) was used as negative control. Indeed, the PLA_2_ is known to be glycosylated with α (1,3)-core fucose *N*-glycans and the Ribonuclease B is bearing high mannose type *N*-glycans (Joao and Dwek, 1993; Lai and Her, 2002). Five μL of the PageRuler Plus Prestained Protein Ladder (Thermo Fisher) was loaded as molecular weight markers. After separation on NuPAGE Bis-Tris gel, proteins were blotted on a nitrocellulose membrane using a semi-dry transfer Thermo Scientific^TM^ Pierce^TM^ Power Blotter. A Ponceau S staining was performed to visualize the efficiency of protein transfer on the membrane.

Western blot was saturated overnight in TBS-T and then incubated with the anti-α(1,3)-core fucose antibody (Agrisera) as a primary antibody at a dilution of 1/5,000 in TBS-T during 2 h at room temperature. After washing, a secondary goat anti-rabbit antibody conjugated with HRP (Sigma) was used at a dilution of 1/30,000 in TBS-T for 1 h at room temperature. Revelation was performed using the ECL west Pico plus kit (Thermo Fisher) according to the manufacturer’s instructions. Exposure time was 1 min.

## Results

### Presence of Fucose in *Phaeodactylum tricornutum* Glycoconjugates

The activated nucleotide-sugar GDP-L-fucose is synthesized in the cytosol from GDP-D-mannose. Bioinformatic analysis revealed that homologs of GDP-D-mannose-4,6-dehydratase and the GDP-4-keto-6-deoxy-D-mannose-3,5-epimerase-4-reductase, the two enzymes of the GDP-L-fucose pathway in eukaryotes, are predicted in the *P. tricornutum* genome (Gügi et al., 2015). Moreover, little amount of fucosylated *N*-glycans has already been described in *P. tricornutum* (Baïet et al., 2011). To investigate whether *P. tricornutum* accumulates other fucose-containing polymers, a monosaccharide composition of an alcohol insoluble fraction (AIR) was carried out by gas chromatography analysis. Fucose was found to represent about 4% of total monosaccharides in this diatom fraction (Table 1), suggesting that the other polysaccharides or glycoconjugates of *P. tricornutum* may contain fucose monomers. The relative proportions of other monosaccharides are consistent with those previously reported (Abdullahi et al., 2006; Gügi et al., 2015). The high percentage of mannose is likely to originate from the major cell wall polysaccharide of *P. tricornutum*, namely sulfated glucuronomannan (Ford and Percival, 1965; Tesson et al., 2009).

**Table 1:** Monosaccharide composition of an alcohol insoluble fraction (AIR) isolated from *P. tricornutum* cells.

Monosaccharide	Rha	Fuc	Xyl	GlcUA	ManUA	Man	Gal	GalUA	Glc
% Relative composition mean ± SE	13.1 ± 0.8	4.0 ± 0.5	13.1 ± 0.4	7.9 ± 0.6	1.1 ± 0.1	46.6 ± 1.0	10.3 ± 0.3	0.8 ± 0.0	3.1 ± 1.1

*Results are expressed in relative percentage (%). Values are the means of monosaccharide quantities determined in triplicate by GC-FID analysis performed on AIR isolated from four independent cell cultures. Rha, rhamnose; Fuc, fucose; Xyl, xylose; GlcUA, glucuronic acid; ManUA, mannuronic acid; Man, mannose; Gal, galactose; GalUA, galacturonic acid; Glc, glucose.*

### *Phaeodactylum tricornutum* Putative Transporter Predicted Protein Sequences Exhibits Strong Amino Acid Identities With Eukaryotic GDP-Fucose Transporters

**FIGURE 1:**
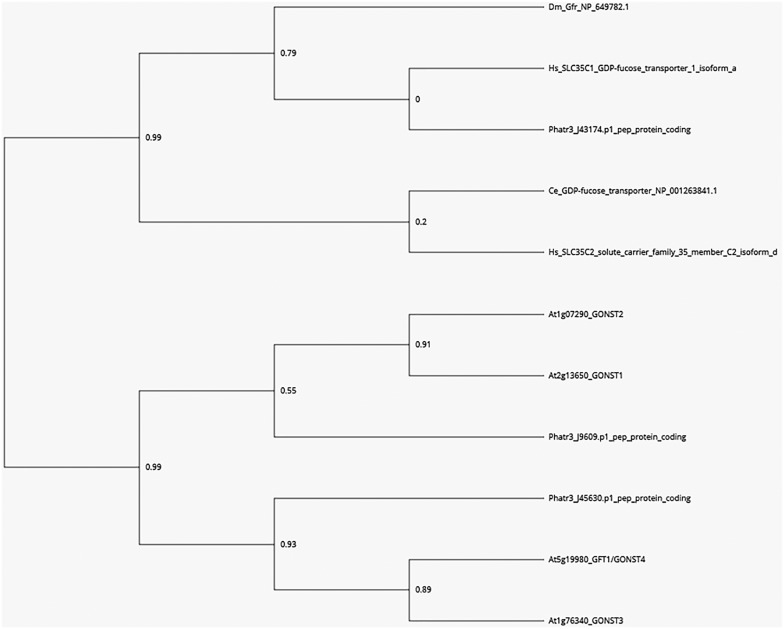
Phylogenetic tree of GDP-L-fucose and GDP-D-mannose transporters from eukaryotes based on Rautengarten et al. (2016) and Ebert et al. (2017). A BLASTP search was carried out in the sequence data of *P. tricornutum* using the GOLGI-LOCALIZED NUCLEOTIDE SUGAR TRANSPORTER GONST1, GONST2, GONST3, and GFT1/GONST4 amino acids sequences of *A. thaliana*. Three encoding sequences corresponding to the Phatr3_J43174, Phatr3_J45630, and Phatr3_J9609 were identified. These *A. thaliana* and *P. tricornutum* sequences were compared to the functionally characterized animal GDP-fucose transporters from *H. sapiens* (NP_060859.4, SLC35C1_GDP-fucose transporter 1 isoform a), *C. elegans* (NP_001263841.1, GDP-fucose transporter), *D. melanogaster* (NP_649782.1, Gfr) and also the *H. sapiens* SLC35C2 (NP_001268386, solute carrier family 35 member C2 isoform d). A phylogenetic analysis and tree were conducted using the *Phylogeny.fr* platform (Dereeper et al., 2008, 2010). Bootstrap values are shown at branch points.

For the synthesis of either polysaccharides or glycoproteins, GDP-L-fucose has to be imported by a specific GDP-L-fucose transporter into Golgi cisternae where the elongation of glycans occurs. In order to search for putative candidates for GDP-sugar transporters in *P. tricornutum*, a BlastP analysis was carried out using the GOLGI-LOCALIZED NUCLEOTIDE SUGAR TRANSPORTER GONST1, GONST2, GONST3, and GFT1/GONST4 amino acids sequences of *A. thaliana* as queries (Baldwin et al., 2001; Handford et al., 2004; Mortimer et al., 2013; Rautengarten et al., 2016). This allows the identification of three protein sequences encoded by the Phatr3_J43174, Phatr3_J45630, and Phatr3_J9609 genes. These *P. tricornutum* sequences were also compared with the functionally characterized GDP-L-fucose transporters from *H. sapiens* (NP_060859.4, SLC35C1_GDP-fucose transporter 1 isoform a), *C. elegans* (NP_001263841.1, GDP-fucose transporter), *D. melanogaster* (NP_649782.1, Gfr), and the *H. sapiens* SLC35C2 (NP_001268386, solute carrier family 35 member C2 isoform d). Phylogenetic analysis shows that the protein encoded by the Phatr3_J43174 is more closely related to the GDP-L-fucose transporter from mammals and *C. elegans* whereas the two other putative GDP-sugar transporters encoded by Phatr3_J45630 and Phatr3_J9609, respectively, are related to the GFT1/GONST4 (Rautengarten et al., 2016) and the GONST 2 and 3, respectively (Figure 1). Due to the fact that (1) the codon usage in *P. tricornutum* is much closer to that of human (Heitzer et al., 2007; Bowler et al., 2008), (2) the cell metabolism from *P. tricornutum* shares common features with both animals and plants (De Martino et al., 2009; Martin-Jézéquel and Tesson, 2012) and (3) its best amino acids sequence homology with the human SLC35C1 (Luhn et al., 2001; Ishida and Kawakita, 2004; Zhang et al., 2012), the protein sequence encoded by the Phatr3_J43174 gene (NCBI accession number: XP_002177476.1) was selected as the most promising GDP-L-fucose transporter candidate in *P. tricornutum*.

### Functional Characterization of the GDP-Fucose Transporter Encoded by the Phatr3_J43174 Gene in *P. tricornutum*

#### The GDP-Fucose Transporter Is Expressed in *P. tricornutum*

**FIGURE 2:**
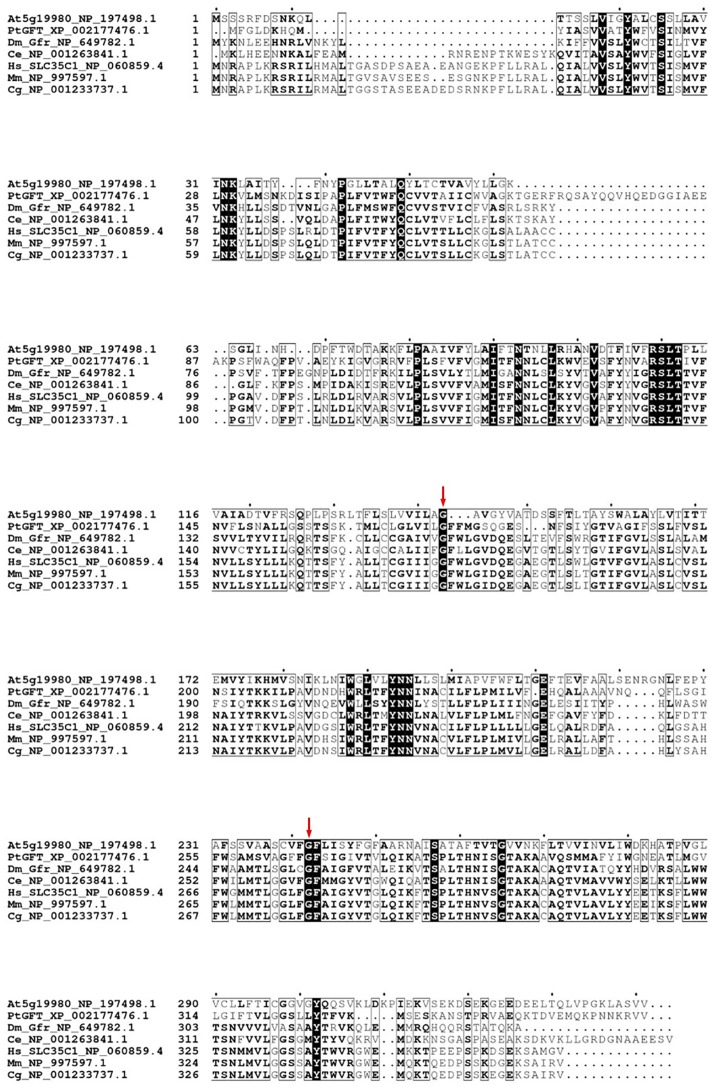
*Phaeodactylum tricornutum* putative GFT (PtGFT) exhibits strong amino acid identities with other eukaryotic GDP-L-fucose transporters. Multiple alignments of GDP-L-fucose transporters protein sequences from *H. sapiens* (NP_060859.4; *SLC*35C1 gene), *M. musculus* (NP_997597.1; *SLC*35C1 gene), *C. griseus* (NP_001233737.1; *SLC*35C1 gene), *C. elegans* (NP_001263841.1; nstp-10 gene), *D. melanogaster* (NP_649782.1; Dm_Gfr gene); *A. thaliana* [NP_197498.1; At5g19980 (GFT1/GONST4) gene], and PtGFT (XP 002177476.1) with the MUSCLE program (https://www.ebi.ac.uk/; Edgar, 2004). The figure was created with the Espript program (http://espript.ibcp.fr/ESPript/ESPript/index.php; Robert and Gouet, 2014). The two red arrows highlight the glycine residues positions 171 and 266, respectively, in the PtGFT_XP_002177476.1 sequence.

Phatr3_J43174 codes for a protein of 360 amino acids in length that is in agreement with the expected length for nucleotide sugar transporters (Ishida and Kawakita, 2004). The candidate protein from *P. tricornutum* is a hydrophobic protein predicted to be a type III membrane protein containing 8–10 membrane-spanning helices as observed for nucleotide-sugar transporters that act as antiporters able to exchange cytosolic nucleotide-sugars for the corresponding nucleotide monophosphate (Supplementary Figure S1). The same topology has previously been described for the *H. sapiens*, *C. elegans*, and *D. melanogaster* GDP-L-fucose transporter (Luhn et al., 2001, 2004; Ishida and Kawakita, 2004; Geisler et al., 2012; Peterson et al., 2013; Rautengarten et al., 2016). The *P. tricornutum* Phatr3_J43174 deduced protein sequence exhibited 46% amino acid identities (with a query coverage of 88%) with the human GDP-L-fucose transporter (Figure 2). Identities from 39 to 45% were observed with GDP-L-fucose transporters from other eukaryotic organisms such as *M. musculus* (NCBI accession number NP_997597.1), *C. elegans* (NCBI accession number NP_001263841.1), *H. sapiens* (NCBI accession number NP_060859.4); *C. griseus* (NCBI accession number NP_001233737.1), or *D. melanogaster* (NCBI accession number NP_649782.1). Only 22% of identity (with a query coverage of 84%) were observed between the *P. tricornutum* and *A. thaliana* (NCBI accession number NP_197498.1) proteins. Furthermore, the putative sequence possesses a conserved *C*-terminal tail which is known to be crucial for Golgi localization and GDP-L-fucose import into the Golgi apparatus (Zhao et al., 2006; Lim et al., 2008; Zhang et al., 2012). Moreover, the two Gly residues (positions 171 and 266, respectively, in the PtGFT_XP_002177476.1; Figure 2) which are required for GDP-L-fucose import in the Golgi apparatus are conserved in the *P. tricornutum* protein candidate (Zhang et al., 2012). Considering the high sequence identities with biochemically characterized GDP-L-fucose transporters, we postulate that this protein is able to import GDP-L-fucose in the Golgi apparatus of *P. tricornutum* and is, accordingly, named *P. tricornutum* GDP-L-fucose transporter (PtGFT) in this paper. To determine whether the PtGFT gene is expressed in *P. tricornutum*, PCR analyses using specific PtGFT primer pairs were performed on cDNA and gDNA prepared from *P. tricornutum* cells and allowed the amplification of specific bands (Supplementary Figure S2). Difference in size of amplified sequences reflected the presence of an intron in the PtGFT candidate gene.

#### The GDP-Fucose Transporter From *P. tricornutum* Is Able to Rescue the Glycoprotein Fucosylation in the CHO-gmt5 Cells

**FIGURE 3:**
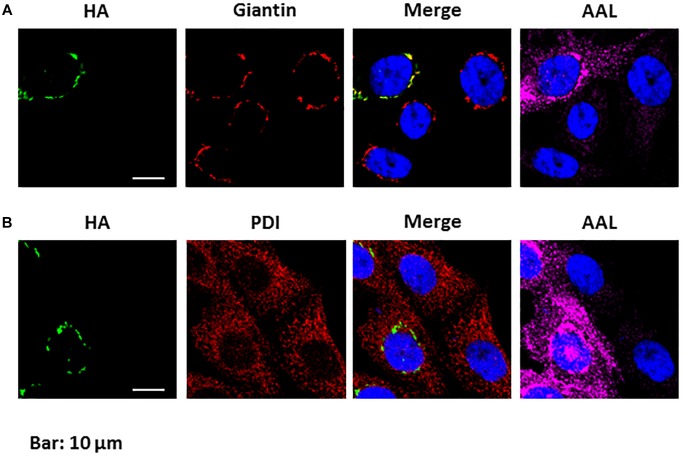
*Phaeodactylum tricornutum* GFT is localized in the Golgi apparatus and is able to rescue protein fucosylation in CHO-gmt5 cells. **(A)** Immunolabelling of HA-tagged PtGFT; Giantin and affinostaining with AAL of CHO-gmt5 cells expressing HA-tagged PtGFT. The merge image between the immunolabelling of HA-tagged PtGFT and the Giantin shows the co-localization of both proteins expressed in the CHO-gmt5 cells. **(B)** Immunolabelling of HA-tagged PtGFT and the PDI ER resident protein, and affinostaining with AAL of CHO-gmt5 cells expressing HA-tagged PtGFT. Note that only CHO cells expressing HA-tagged PtGFT exhibit an AAL staining. Hoechst: nucleus labeling of living CHO cells. The indicated scale bar is 10 μm for all the images of the figure.

**FIGURE 4:**
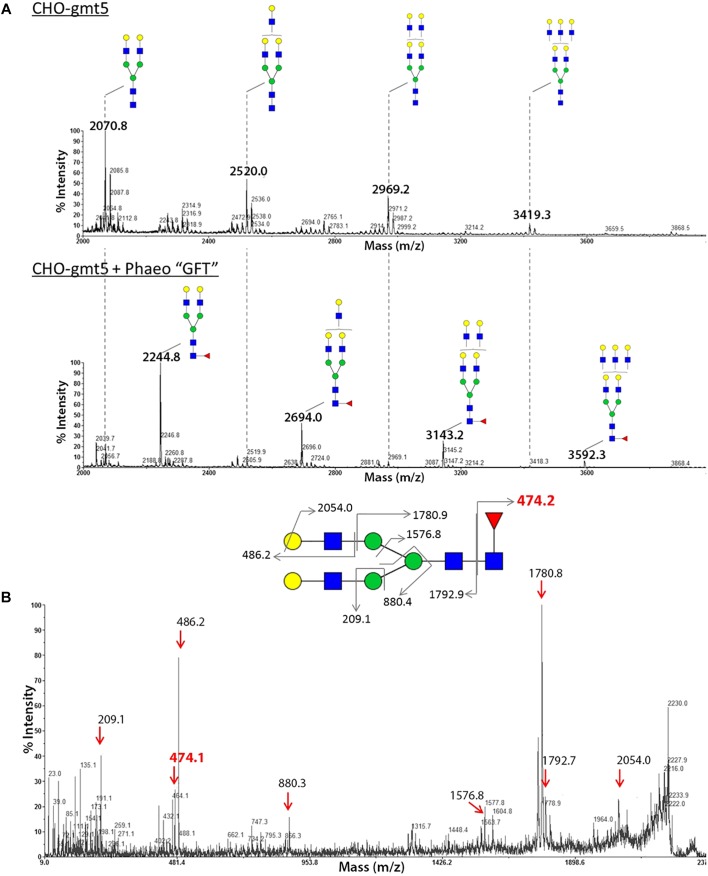
Analysis of the *N*-glycan profile of proteins from CHO gmt5 cells expressing PtGFT revealed fucosylated *N*-glycans. MALDI-TOF MS profiles of permethylated *N*-glycans isolated from total proteins of **(A)** CHO-gmt5 cell line before (CHO-gmt5) or after complementation with the PtGFT (CHO-gmt5 + Phaeo“GFT”). **(B)** MS^2^ fragmentation pattern of a biantennary *N*-glycan at *m/z* 2244 demonstrating that the fucose residue is linked to the proximal GlcNAc residue. 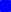 N-acetylglucosamine, 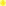 galactose, 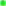 mannose, 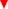 fucose.

To investigate its cell localization and its biochemical function, a *N*-terminal HA-tagged version of the PtGFT was transiently expressed in the CHO-gmt5 mutant cell line that is devoid of endogenous GDP-L-fucose transporter (Zhang et al., 2012; Haryadi et al., 2013). GDP-L-fucose transporter has been shown to be localized in the Golgi apparatus to supply this compartment with activated fucose (Luhn et al., 2001). To ascertain the localization of PtGFT in the Golgi apparatus, CHO-gmt5 cells transiently expressing the HA-tagged PtGFT were examined using immunofluorescence microscopy. PtGFT sub-cellular localization pattern was compared to that of Giantin, a Golgi marker and that of the Protein Disulfide Isomerase, an ER-resident soluble protein. As shown in Figure 3A,B, HA-tagged PtGFT clearly co-localized with Giantin but not with Protein Disulfide Isomerase (PDI in the Figure 3B), indicating that PtGFT is efficiently targeted to the Golgi membrane in the CHO-gmt5 cells. To claim the capacity of PtGFT to transport GDP-L-fucose in the complemented mutant, CHO-gmt5 cells transiently expressing PtGFT were affinodetected with Aleuria Aurantia Lectin (AAL), a fucose-specific lectin exhibiting a strong affinity toward core fucose and Lewis-X epitope on *N*-linked glycans (Bergstrom et al., 2012; Zhang et al., 2012). As shown in Figure 3B, only cells expressing PtGFT exhibit AAL staining, suggesting that the diatom transporter candidate is able to rescue fucosylation of proteins in the PtGFT complemented CHO-gmt5 mutant cells. Such a result also confirmed the Golgi localization of the PtGFT. To further confirm that PtGFT is able to rescue a wild-type fucosylation of endogenous proteins, *N*-glycosylation of proteins in the CHO-gmt5 complemented with the PtGFT was investigated. Proteins from both non-complemented and complemented CHO-gmt5 cell lines were isolated. *N*-linked glycans to proteins were then released by PNGase F treatment, permethylated and then analyzed by MALDI-TOF/TOF mass spectrometry (Figure 4A). As expected, *N*-glycans isolated from CHO-gmt5 were mostly asialo, afucosylated galactosylated bi- and tri-antennary *N*-glycans. In PtGFT complemented line, similar species were detected in the *N*-linked glycan profile with a shift of 174 mass units assigned to an additional permethylated deoxyhexose (fucose) residue. Furthermore, MS^2^ fragmentation pattern of a galactosyl biantennary glycan clearly shows a fragment ion at *m/z* 474 confirming the core fucosylation of *N*-linked glycans in the PtGFT CHO-gmt5 complemented line (Figure 4B). Taken together, both AAL staining and mass spectrometry *N*-glycan profiling of CHO-complemented cells demonstrated that expression of PtGFT in CHO-gmt5 cell line is able to rescue the fucosylation of proteins by complementing the lack of endogenous GDP-L-fucose transporter in the CHO mutant line. This demonstrates the capacity of PtGFT to import the GDP-L-fucose within the Golgi apparatus where the *N*-glycan fucosylation takes place.

### Functional Characterization of the Fucosyltransferases in *P. tricornutum*

#### Specific Features of Putative Fucosyltransferases in *P. tricornutum*

**FIGURE 5:**
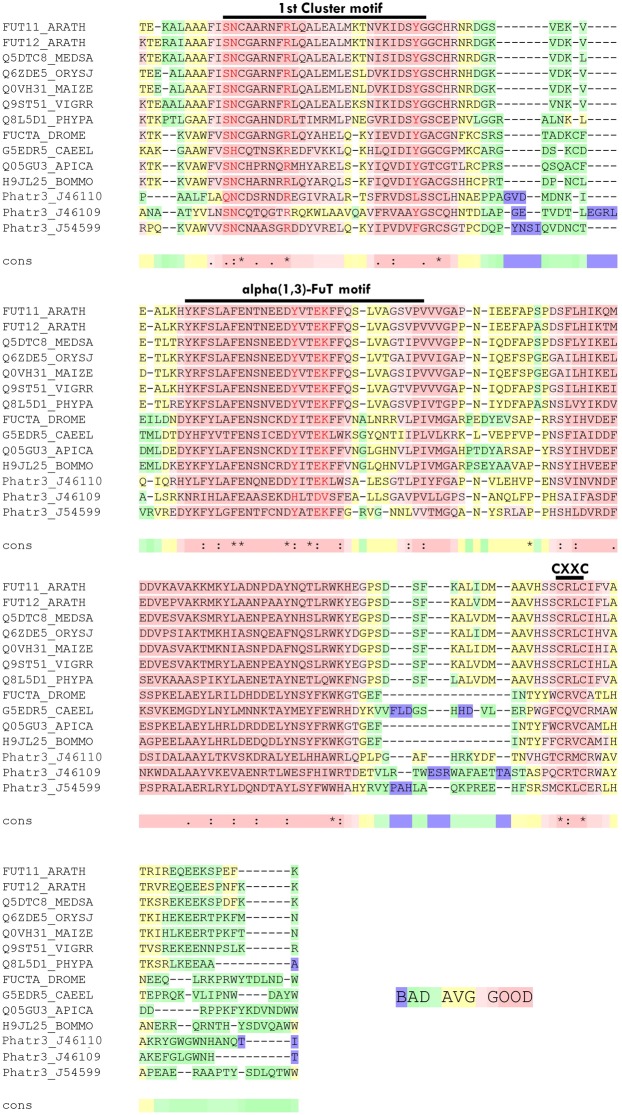
*Phaeodactylum tricornutum* putative FuT exhibits strong amino acid identities with α(1,3)-fucosyltransferases. Amino acids sequences comparison with the T-coffee program (http://tcoffee.crg.cat/, Di Tommaso et al., 2011) of the C-terminal GT10 domains of the three putative fucosyltransferases encoded, respectively, by the Phatr3_J46109, Phatr3_J46110 and Phatr3_J54599 genes from *Phaeodactylum tricornutum* with biochemically characterized α(1,3)-fucosyltransferases from *Arabidopsis thaliana* (FUT11_ARATH and FUT12_ARATH), *Medicago sativa* (Q5DTC8_MEDSA), *Oryza sativa* (Q6ZDE5_ORYSJ), *Zea mays* Q0VH31_MAIZE), *Vigna radiata* (Q9ST51_VIGRR), *Physcomitrella patens* (Q8L5D1_PHYPA), *Drosophila melanogaster* (FUCTA_DROME), *Caenorhabditis elegans* (G5EDR5_CAEEL), *Apis mellifera* (Q05GU3_APICA) and *Bombyx mori* (H9JL25_BOMMO). The graphic output reflects the level of consistency of the alignment of a considered residue (from blue/green: badly or poorly supported to pink which corresponds to strongly supported). Conserved motifs of the GT10 domain are indicated on the top of the alignment.

Three FuT have been predicted in a preliminary investigation of the *P. tricornutum* genome (Baïet et al., 2011). These predicted proteins of 798, 707, and 481 amino acids are encoded, respectively, by the Phatr3_J46109, Phatr3_J46110, and Phatr3_J54599 genes. The three putative fucosyltransferases have been described to possess structural features characteristic for the CAZy family GT10, family to which belongs the FuT(Baïet et al., 2011). The three protein sequences exhibit Pfam GT10 domains. Only one GT domain is present in the C-terminal part of Phatr3_J46109 and Phatr3_J46110 as observed for plant α(1,3)-FuT (Both et al., 2011), whereas two domains are predicted for Phatr3_J54599 as reported for invertebrate α(1,3)-FuT by Pfam analysis (El-Gebali et al., 2019). The amino acid sequences corresponding to the Glyco_Transf_10 domains (described in the NCBI Conserved Domain Database) of the putative *P. tricornutum* FuT were compared to those of various characterized FuT from plant and invertebrates by using the T-coffee multiple sequence alignment server^3^ (Di Tommaso et al., 2011). All putative FuT present the CXXC motif located on the C-terminal end of the proteins which is well known to be essential for the enzymatic activity as it is involved in the formation of disulfide bridges which favor good folding of the FuT (Holmes et al., 2000). Additional conserved domains named “1st cluster” and “α1,3-FuT motif” are also present in the three candidates. These domains are described to be involved in the binding to the donor substrate which is the GDP-L-fucose for fucosyltransferase activity (Both et al., 2011). Moreover, the amino acids which are indicated in red in the Figure 5 are conserved in the *P. tricornutum* putative FuT. These residues have been demonstrated by directed mutagenesis to be involved in the binding to the acceptor substrate. Based on RNA-seq data recently reported in Ovide et al. (2018), mainly Phatr3_J46109 and Phatr3_J54599 are expressed in the three morphotypes of *P. tricornutum* (fusiform, triradiate, and oval morphotypes).

#### FuT54599 Is Active and Localized in the Golgi Stacks

**FIGURE 6:**
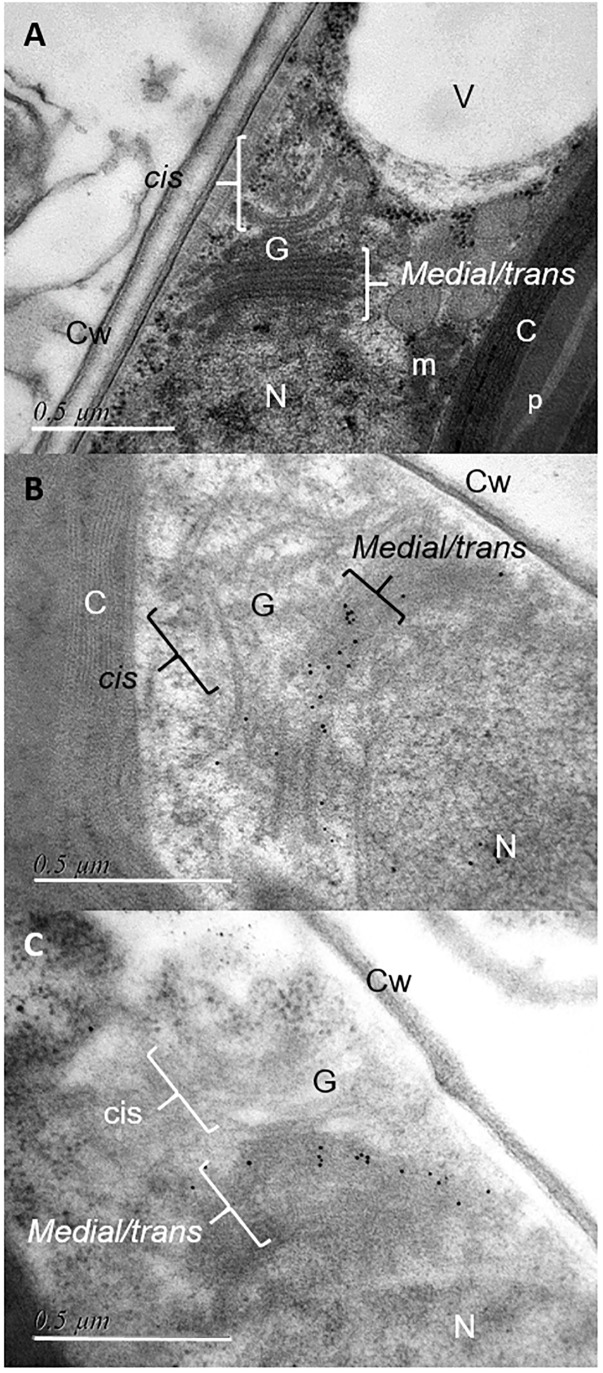
Immunocytochemical localization of V5-tagged FuT54599 and V5-tagged GnT I in the Golgi apparatus of *P. tricornutum.* Transmission Electron micrographs of HPF/FS *P. tricornutum* cells which were embedded in LRW resin. **(A)** View showing a Golgi apparatus of *P. tricornutum* oriented as previously described for algae cells in Donohoe et al. (2007, 2013). **(B)** Localization of the V5-tagged GnT I in the *medial-trans* Golgi cisternae of *P. tricornutum* Golgi apparatus after immunolabelling with a mouse anti-V5 antibody used as a primary antibody (dilution 1/20) and a secondary antibody coupled to 10 nm gold beads (dilution 1/20) and then contrasted with uranyl acetate and lead citrate. **(C)** Localization of the V5-tagged FuT54599 in the *median-trans* Golgi cisternae of *P. tricornutum* Golgi apparatus after immunolabelling with a rabbit anti-V5 antibody used as a primary antibody (dilution 1/20) and a secondary antibody coupled to 10 nm gold beads (dilution 1/20) and then contrasted with uranyl acetate and lead citrate. N, nucleus; G, Golgi apparatus with *cis* and medial/*trans* cisternae; V, vacuole; m, mitochondria; p, pyrenoid; C, chloroplast; Cw, cell wall. Scale bar: 0.5 μm. Please refer to the Supplementary Figure S4 for negative controls.

**FIGURE 7:**
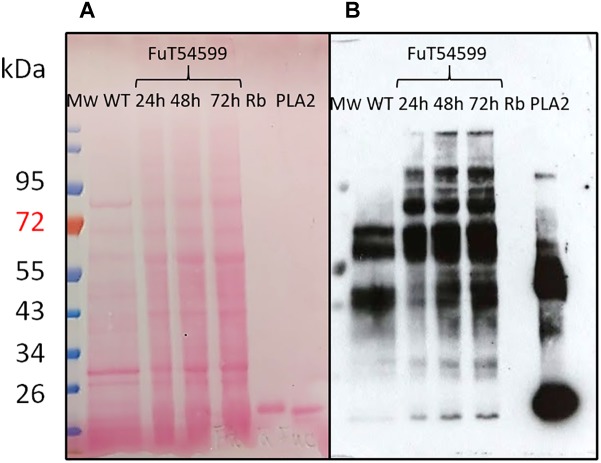
*N*-glycans bearing the α(1,3)-fucose epitope are increasing in *P. tricornutum* cells expressing the Golgi resident V5-tagged FuT54599. Western Blot analysis of total protein extracts from WT (lane 1) and *P. tricornutum* cells expressing the V5-tagged FuT54599 using a specific core α(1,3)-fucose antibody. Total proteins have been extracted from *P. tricornutum* cells expressing the V5-tagged FuT54599 24 h after induction (lane 2); 48 h after induction (lane 3); 72 h after induction (lane 4). The 15 kDa Ribonuclease B (Rb) was used a negative control (lane 5) and the 14.5 kDa Phospholipase A2 (PLA2) was used as a positive control (lane 6). In parallel, molecular weight (Mw) markers (PageRuler Plus Prestained Protein Ladder, Thermo Fisher) are reported and expressed in kDa. **(A)** Ponceau red staining of the membrane. **(B)** Revelation with the anti-α(1,3)-fucose antibody (Agrisera).

The coding sequences of the three putative FuT were cloned and expressed in *P. tricornutum* in fusion to a C-terminal V5 tag which allows detection of the recombinant glycosyltransferase as previously described for the heterologous expression of the *N*-acetylglucosaminyltransferase I (GnT I) from *P. tricornutum* within the CHO Lec1 mutant cells (Baïet et al., 2011). Clones expressing the V5-tagged fucosyltransferase respective genes were selected by PCR and RT-PCR analyses. Only *P. tricornutum* lines expressing the V5-tagged version of the Phatr3_J54599 fucosyltransferase candidate, called FuT54599 from now, were positive and further studied. The other clones transformed with the Phatr3_J46109 and Phatr3_J46110 genes, respectively, were negative by RT-PCR and later by Western blot analyses. In contrast, analyses by western blot using a V5 specific antibody of microsomal fractions extracted from the transformed lines expressing the V5-tagged FuT54599 revealed, 24 h after induction a specific band above 60 kDa, suggesting that the FuT54599 might be localized within the Golgi apparatus in *P. tricornutum* (Supplementary Figure S3). In order to confirm this result and to precisely localize the FuT54599 at the sub-cellular level, Transmission Electron Microscopy (TEM) coupled to immuno-gold labeling using antibodies directed against the V5 epitope was used on high pressure frozen samples (Figure 6). Such a technique has already been employed to localize glycosyltransferases within Golgi stacks in plant cells (Chevalier et al., 2010). *P. tricornutum* cell lines overexpressing a V5-tagged of its endogenous GnT I, which is a Golgi-resident transferase involved in the *N*-linked glycans modifications as previously reported (Baïet et al., 2011), have been studied in parallel to the FuT54599. The excellent structural preservation of the different membrane system in high pressure frozen *P. tricornutum* cells allows us to orientate the Golgi apparatus. Indeed, as described in Donohoe et al. (2007, 2013), *cis* cisternae exhibit a much lighter luminal staining and possess a thicker lumen as compared to the medial cisternae. The *trans*-Golgi cisternae is presenting a collapsed central luminal domain and swollen margins. As illustrated in Figure 6, the V5-tagged FuT54599 was found to be preferentially located in the medial/*trans* Golgi cisternae. A similar immunogold labeling was observed for the V5-tagged GnT I protein. In addition, western blot analysis using a specific anti-α(1,3)-fucose antibody on total protein extract from *P. tricornutum* revealed an increase of the level of α(1,3)-fucose epitopes associated with proteins of *P. tricornutum* lines overexpressing the V5-tagged FuT54599 as compared to the wild-type cells (Figure 7).

## Discussion

In the context of the production of biopharmaceuticals dedicated to human therapy in *P. tricornutum*, a comprehensive understanding of its protein glycosylation is crucial. Core α(1,3)-fucose have been detected on glycans *N*-linked to proteins of *P. tricornutum*. This glyco-epitope may induce immune responses in humans after injection of a biopharmaceutical produced in this diatom. As a consequence, inactivation of genes encoding key enzymes of the fucosylation machinery will likely be required, taking advantage of recent progresses that have been achieved in *P. tricornutum* to develop genome editing tools such as TALEN and CRISPR/Cas9 (Daboussi et al., 2014; Nymark et al., 2016; Allorent et al., 2018; Kroth et al., 2018; Serif et al., 2018; Slattery et al., 2018; Stukenberg et al., 2018).

Fucosylation of glycoproteins starts by the cytosolic biosynthesis of GDP-L-fucose, its import into the Golgi apparatus and finally its transfer onto the glycoproteins within Golgi cisternae. With regard to the import in the Golgi apparatus of the fucose-activated nucleotide, we identified a putative GDP-L-fucose transporter exhibiting high sequence identity with well-characterized GDP-L-fucose transporters. When expressed in CHO-gmt5 mutant lacking endogenous GDP-L-fucose transporter activity, the PtGFT candidate is efficiently addressed to Golgi membranes and is able to rescue the fucosylation of proteins in the CHO-gmt5 mutant cell line, demonstrating that the cDNA sequence registered under the NCBI accession number KT737477 codes a functional Golgi resident *P. tricornutum* transporter which is at least able to import GDP-L-fucose. This suggests that molecular mechanisms controlling the targeting and nucleotide-sugar import are conserved between mammals and microalgae. To the best of our knowledge, this is the first diatom nucleotide-sugar transporter characterized to date. Two other putative GDP-sugar transporters are also predicted in *P. tricornutum* genome. We postulate that they may be involved in the D-mannose import, another abundant monosaccharide detected in this diatom that is also activated in the cytosol by coupling to GDP. In this work, we identified a transporter from *P. tricornutum* which is able to transport at least the GDP-L-fucose. However, based on the methodology used in this study (complementation of the CHO-gmt5 mutant) and on the monosaccharide composition of AIR from *P. tricornutum* which contained more than 44% of mannose, we cannot completely rule out that the transporter from *P. tricornutum* is not able to transport other nucleotide sugars like the GDP-D-mannose as well. Indeed, in *A. thaliana*, initial studies have established that GONST1 is within Golgi stacks and can functionally complement the yeast vanadate resistance glycosylation GDP-D-mannose transporter mutant (Baldwin et al., 2001; Handford et al., 2004). However, years later, this GONST1 transporter was described to be able to transport 4 different GDP-sugars *in vitro* (Mortimer et al., 2013). Moreover, mutation in the GONST1 only alters the glycosylation of the glycosylinositol phosphoceramide, even if GDP-D-mannose is used in the biosynthesis of multiple glycoconjugates in the Golgi apparatus. Therefore, future work would be necessary to characterize the function of the other putative GDP-sugar transporters from *P. tricornutum*. In addition, it would be interesting to evaluate the physiological impact of the GFT inactivation in *P. tricornutum* and demonstrate whether the fucose transport is essential in the diatom as it is in *A. thaliana* and humans. Indeed, *A. thaliana gonst1* mutants are dwarfed and developed spontaneous leaf lesions (Mortimer et al., 2013). Moreover, previous alteration of the *mur1* gene (encoding for an isoform of the GDP-D-mannose-4,6-dehydratase) in *A. thaliana* demonstrated that the availability of GDP-L-fucose is critical for normal plant development and cell wall structure (Bonin et al., 1997; Rayon et al., 1999; Reuhs et al., 2004). In humans, missense mutations in the GDP-fucose transporter cDNA of patients suffering from a congenital disorder of glycosylation type IIc cause many symptoms including mental retardation, short stature, facial stigmata, and recurrent bacterial peripheral infections with persistently elevated peripheral leukocytes (Lübke et al., 2001).

Three putative FuT are also predicted in *P. tricornutum* genome. They exhibit Pfam GT10 domains as observed for α(1,3)-FuT of plants and invertebrates (Wilson et al., 2001; Both et al., 2011). We attempted to overexpress the three FuT candidates as fusion proteins containing a C-terminal V5 tag in *P. tricornutum*. However, only the V5-tagged FuT54599 was detected. A monoexonic gene encodes this protein. Moreover, this protein is expected to be 481 amino acids long which is in agreement with core α(1,3)-fucosyltranferases characterized earlier in plants and invertebrates (Wilson et al., 2001; Paschinger et al., 2004). When looking at the topology, the FuT54599 is the only candidate which is clearly predicted to be a type II protein. Such topology has been observed for Golgi-resident proteins and especially glycosyltransferases (Czlapinski and Bertozzi, 2006). This includes a short *N*-terminal cytosolic tail, a transmembrane domain and consequent catalytic domain which is exposed in the lumen of the Golgi apparatus (Czlapinski and Bertozzi, 2006). The fact that the FuT54599 is a Golgi-resident protein has been confirmed in this work through the localization of the FuT54599 by immunogold-electron microscopy. This result is pioneer as so far, the sub-cellular organization in microalgae of Golgi enzymes involved in glycans and glycoconjugates biosynthesis has not been investigated. The V5-tagged FuT54599 was found to be mainly located in the medial/*trans* Golgi (Figure 6A). Similar sub-cellular localization was observed for the V5-tagged GnT I, a Golgi-resident transferase involved in the maturation of *N*-linked glycans (Baïet et al., 2011). These data suggest that GnT I and the putative α(1,3)-FuT are localized within specific Golgi cisternae. Localization in specific cisternal subtypes has been reported in mammals and land plants (Berger and Hesford, 1985; Chevalier et al., 2010; Schoberer and Strasser, 2011). This sub-cellular organization of enzymes within Golgi stacks is believed to control the step-by-step maturation of glycans *N*-linked to proteins along the secretory pathway. We postulate that such a compartmentation of Golgi enzymes would also occur in the microalgae *P. tricornutum.* In a context of the optimization of the *N*-glycosylation of a microalgae-made biopharmaceuticals, glyco-engineering strategies could benefit from such a compartmentation of Golgi enzymes by expressing chimaeric transferases targeted for optimal activity to a specific Golgi cisternae as previously reported for plants (Vézina et al., 2009).

## Author Contributions

MB conceived and supervised the study. MB, M-CK-M, and PZ designed the experiments. CP, CB, BG, PZ, CW, GT, CO, and AM performed the experiments. ZS provided CHO-gmt5 mutant cell line. MB, M-CK-M, PZ, CP, CB, AD, CO, and BG analyzed the data. PL, MB, PZ, M-CK-M, and BG wrote the manuscript. All authors read and agreed on the submission of the manuscript.

## Conflict of Interest Statement

The authors declare that the research was conducted in the absence of any commercial or financial relationships that could be construed as a potential conflict of interest.

## Footnotes

1 http://tcoffee.crg.cat/

2 https://phyd3.bits.vib.be/index.html

3 http://tcoffee.crg.cat/
