# Supplementary material for: Characterization of a GDP-Fucose Transporter and a Fucosyltransferase Involved in the Fucosylation of Glycoproteins in the Diatom Phaeodactylum tricornutum
Source: Front Plant Sci. 2019 May 21;10:610. doi: 10.3389/fpls.2019.00610 (PMC6536626; doi:10.3389/fpls.2019.00610)
Supplement: Supplementary file 1 [file Table_1.DOCX]

Supplementary Material

# Supplementary Figures

## Supplementary Figure 1


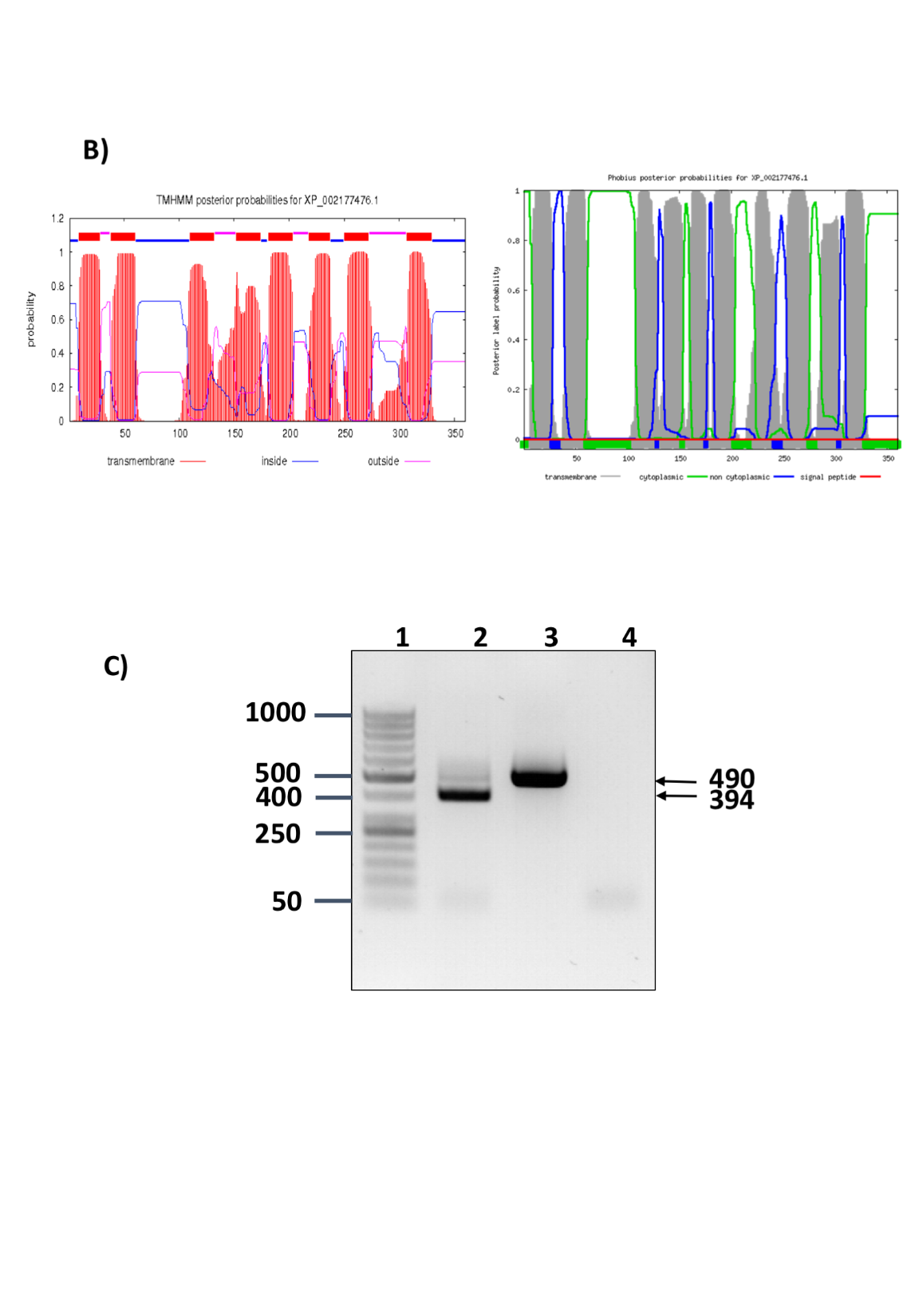


**Supplementary Figure 1:** **PtGFT is predicted to be a transmembrane protein in agreement with the expected topology described for the nucleotide sugar transporters.** Prediction of the transmembrane helices with the TMHMM Server v 2.0 (<http://www.cbs.dtu.dk/services>) (right panel) and Phobius Server (<http://phobius.sbc.su.se/>) (left panel)

## Supplementary Figure 2


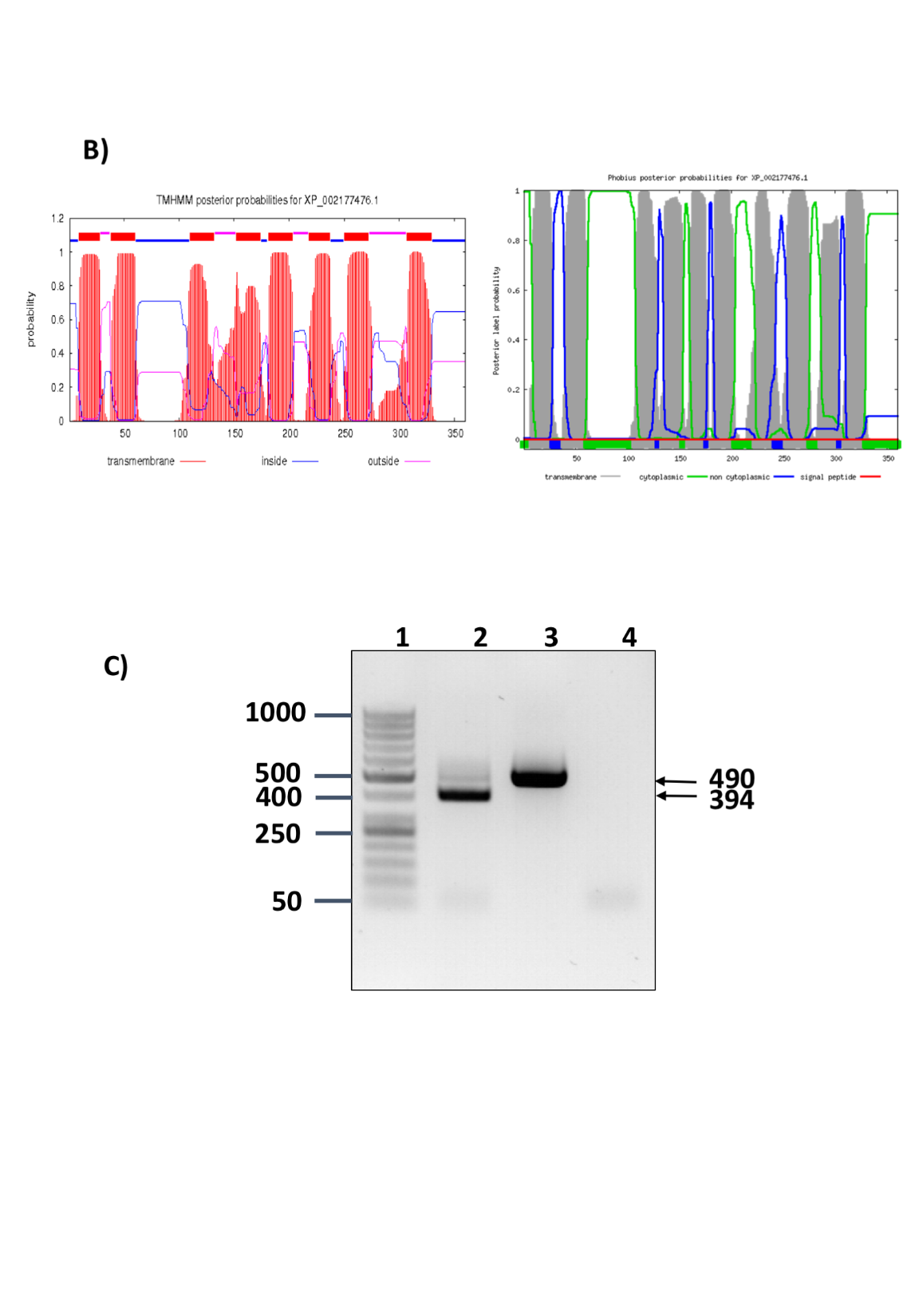


**Supplementary Figure 2:** **PtGFT is expressed in the diatom cells.** Analysis of the PtGFT gene expression by PCR amplification using a specific primer pair as described in the material and methods section. Lane 1: Ladder in bp (GeneRuler 50 bp DNA Ladder, Thermo Fisher Scientific); Lane 2: cDNA; lane 3: gDNA; lane 4: water used as a negative control.

**
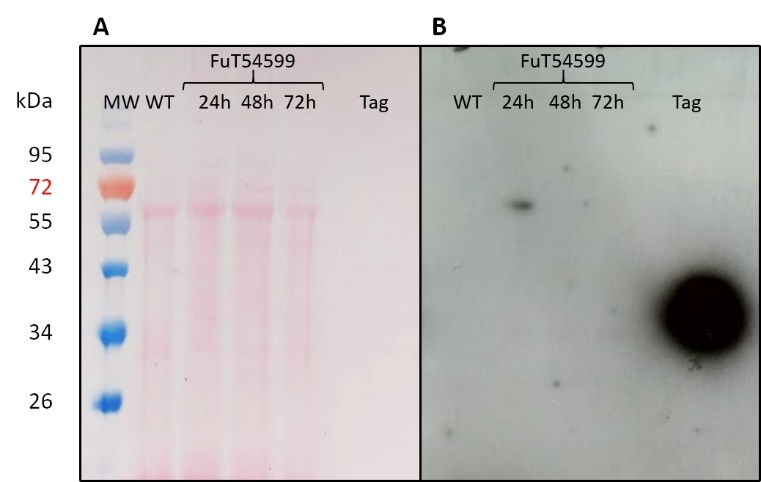
**

**Supplementary Figure 3:** **The V5-tagged FuT54599 is overexpressed 24h after induction in the membrane fraction of *Phaeodactylum tricornutum*.** Western Blot analysis of the membrane fraction proteins from the WT (lane 1) and *P. tricornutum* cells expressing the V5-tagged FuT54599 using a specific V5-tag antibody (lane 2 to 4). Proteins from the membrane fraction have been extracted from the *P. tricornutum* cells expressing the V5-tagged FuT54599 24h after induction (lane 2); 48h after induction (lane 3); 72h after induction (lane 4) and a V5-tagged protein expressed in *E. Coli* (Abcam) called Tag used a positive control (lane 6). In parallel, molecular weight markers (PageRuler Plus Prestained Protein Ladder, Thermofisher) are reported and expressed in kDa. Panel A: Ponceau red staining of the membrane. Panel B: Revelation of the membrane using the specific rabbit anti-V5 antibody (Invitrogen) as described in the material and method section.

**
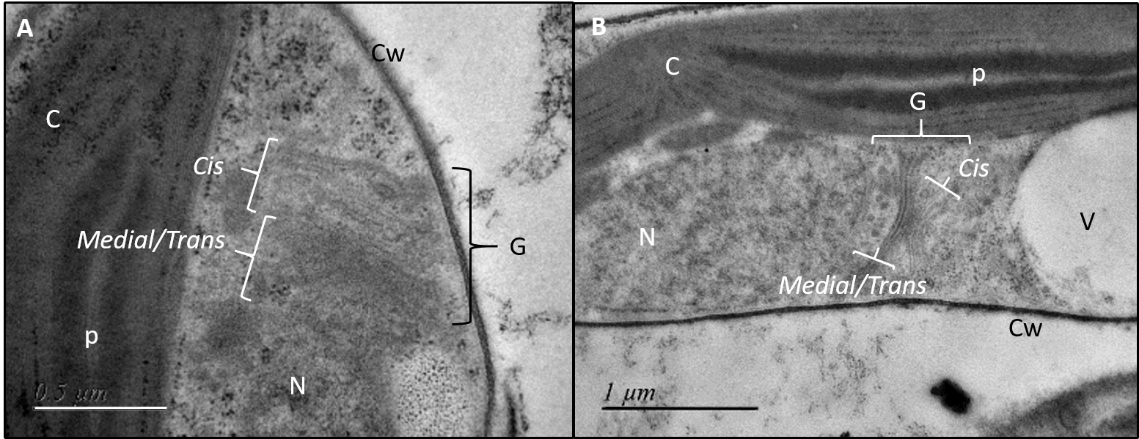
**

**Supplementary Figure 4: Negative controls for the immunocytochemical localization of V5-tagged FuT54599 and V5-tagged GnT I in the Golgi apparatus of *P. tricornutum* illustrating the specificity of the labelling with the anti-V5-tag antibody*.***

Transmission Electron micrographs of HPF/FS *P. tricornutum* cells expressing the V5-tagged GnT I (A) and V5-tagged FuT54599 (B) which were embedded in LRW resin. Views showing a Golgi apparatus of *P. tricornutum* oriented as previously described for algae cells in (Donohoe et al., 2007; Donohoe et al., 2013) after immunolabelling with the secondary antibody coupled to 10 nm gold beads (dilution 1/20) and then contrasted with uranyl acetate and lead citrate. N: nucleus; G: Golgi apparatus with the Cis and Medial/trans cisternae; p: pyrenoid; C: chloroplast. Scale bar: 0.5 µm and 1 µm respectively.

**
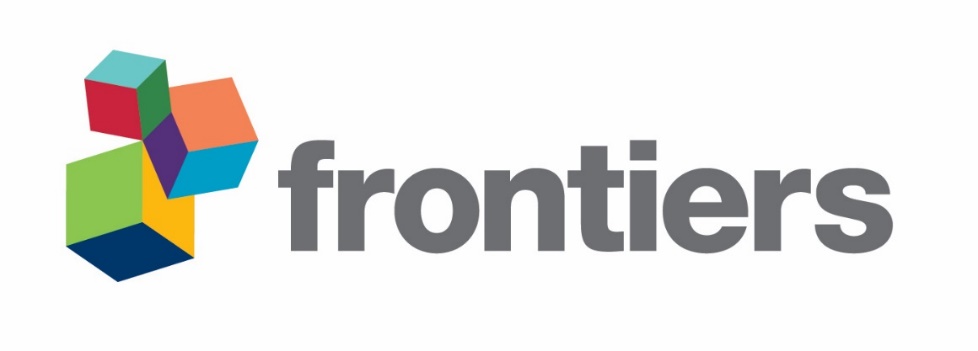
**
